# Fetal Cell Based Prenatal Diagnosis: Perspectives on the Present and Future

**DOI:** 10.3390/jcm3030972

**Published:** 2014-09-03

**Authors:** Morris Fiddler

**Affiliations:** DePaul University and Insight Medical Genetics, LLC 680 N, Lake Shore Drive Chicago, IL 60611, USA; E-Mail: mfiddler@insightmedicalgenetics.com

**Keywords:** fetal cells, fetal cells in maternal circulation, non-invasive prenatal diagnosis, prenatal diagnostics, transcervical retrieval of fetal cells, fetal cells from maternal blood

## Abstract

The ability to capture and analyze fetal cells from maternal circulation or other sources during pregnancy has been a goal of prenatal diagnostics for over thirty years. The vision of replacing invasive prenatal diagnostic procedures with the prospect of having the entire fetal genome in hand non-invasively for chromosomal and molecular studies for both clinical and research use has brought many investigators and innovations into the effort. While the object of this desire, however, has remained elusive, the aspiration for this approach to non-invasive prenatal diagnosis remains and the inquiry has continued. With the advent of screening by cell-free DNA analysis, the standards for fetal cell based prenatal diagnostics have been sharpened. Relevant aspects of the history and the current status of investigations to meet the goal of having an accessible and reliable strategy for capturing and analyzing fetal cells during pregnancy are reviewed.

## 1. Introduction

The use of fetal cells derived from maternal circulation or other sources obtained non-invasively is the “holy grail” of prenatal diagnostics. Although maternal-fetal trafficking of cells has been recognized since the end of the 19th century [1] and subsequently confirmed by many investigators (e.g., [2,3,4,5]), the intense effort to convert this phenomenon into a clinically useful means of prenatal diagnosis has gained momentum over the past thirty years. The anticipated advantages of this strategy have always been compared to the standards set by chorionic villus sampling (CVS) and amniocentesis, *i.e.*, a means to provide a direct source of fetal genomic DNA for chromosome or genotype analyses that provides diagnostic accuracy, reliability, and completeness without the procedural risk or reluctance of a prospective mother to undergo an invasive procedure.

**Figure 1:**
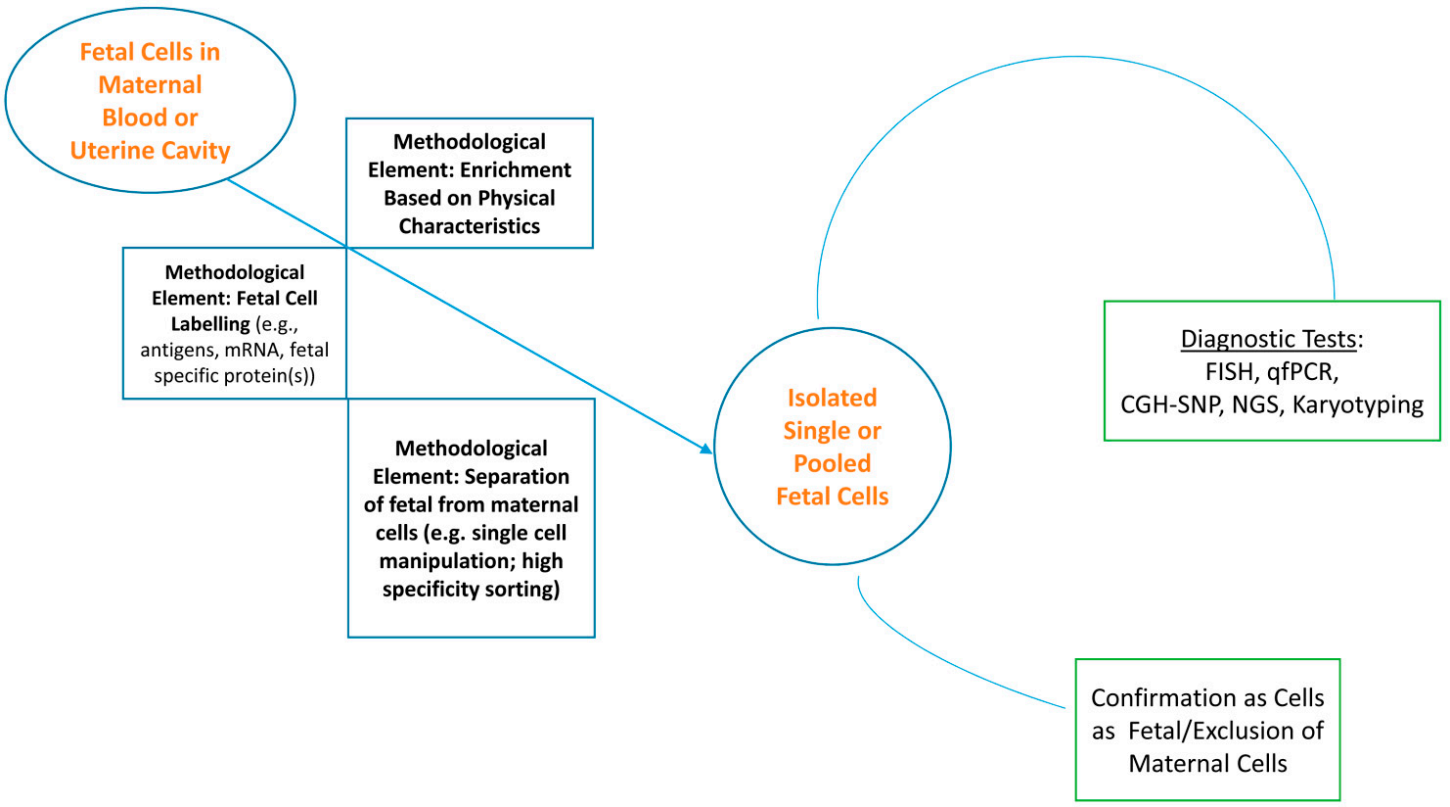
Schema for the capture and analysis of fetal cells for prenatal diagnostics.

The strategies for capturing fetal cells have, to a great extent, been derived from a logic that starts with the premise that the fetal cell is proportionately very rare—approximately one in a billion—in the context of the mother’s erythrocytes as well as her diverse complement of background cells. It then assumes that ultimately the fetal cell must be isolated as a pure population for analysis. A schematic workflow and the methodological elements that are likely to realize that goal are captured in of Figure 1. Enrichment, identification of the cell as distinctly fetal, capture, and diagnostic analysis have been the elements of that logic in one permutation or another. While there are still decision points regarding the sequencing of the steps that will presumably be worked out empirically, the apparent essentials of a strategy have been described in reviews and research reports repeatedly, with relatively minor variations, for approximately 30 years. These have been, to a good extent, modified and/or improved by the advantages of technological innovations [3,4,5,6,7,8,9]. Overall, however, the molecular technology to provide a wide scope of genomic analyses has arrived but the front end delivery of fetal cells remains a link to be strengthened.

Noninvasive prenatal screening and diagnosis based on fetal cells in maternal blood or on placental cells present in the cervix, despite many years of study, have yet to be established as clinically acceptable approaches assessing fetal well-being. Indeed, the presumed advantages of non-invasive prenatal diagnostics using fetal cells over invasive procedures are encountering a significant challenge to their attractiveness as the analysis of cell-free DNA in maternal circulation [10,11,12,13,14] has rapidly gained attention. Taken together, the future landscape of non-invasive prenatal screening and diagnostics could hardly be richer with promise.

## 2. Current Status of Fetal Cell Based Non-Invasive Prenatal Diagnosis

There are at least three perspectives that seem to emerge in assessing the current status. The first is a hybrid of pessimism with patience—while the pursuit of a reliable, consistent, and efficient means for capturing fetal cells in maternal blood has generated scores of publications and stimulated the imaginations of many investigators, the outcomes to date are simply summarized—there is not yet a protocol or technology for a beginning-to-end process for clinical use. Though the number of publications on the subject has diminished dramatically in recent years, the desire to arrive at a functional if not elegant answer to most, if not all, the drawbacks of other screening or diagnostic approaches persists. In 2003, Jackson [7] reviewed the status of fetal-cell based prenatal diagnostics. He concluded that the research to that point had not yet had any consistent success and that significant work lie ahead to develop and validate a strategy and accompanying technology to recover and assay the DNA from fetal cells. Other reviews since then have reached the same conclusion.

This leads to a second perspective on assessing the status of fetal cells for NIPD—that the status as well as the existence of sufficient and verifiable data for a viable methodology to capture fetal cells during pregnancy is really only known to a small handful of people who work within the privacy of corporate based research and development and their advisors. All are held close by non-disclosure agreements, as they seek to protect findings with the barriers of intellectual property and patent laws in the pursuit of an eventual and substantial commercial payoff. This has been the direction manifested in the successes of aneuploidy screening by analysis of cell free DNA from the fetal genome in maternal circulation. The cooperative and sometimes coordinated efforts of independent investigators supported by publicly funded grants and initiative has been subsumed by privately funded interests and entrepreneurial strategies. Consequently, success in creating a clinically applicable system is perhaps close at hand but, then again, perhaps not.

A third perspective is related to the first in that there is always room for new insights and applications of technology developed in other areas, fields, and disciplines that may provide the breakthroughs to bring the use of fetal cells into clinical use of prenatal diagnostics.

## 3. Revisiting the Pursuit

The reasons to continue the pursuit of fetal cells as the presumed best source of information into the genetic status of a fetus have not changed over the approximately 30 years of this research: Fetal cells offer the capacity to provide diagnostic level assessment of chromosomal, single gene, and, eventually, polygenic aberrations to women and couples of any age without the procedural risks that accompany invasive procedures. In addition, just as importantly if not more so, this testing has the potential to provide reassurance to the 95%–97% of women with unaffected pregnancies [15,16,17,18].

There are two sources of fetal cells during pregnancy that have received the primary attention of investigators: the maternal circulation and the uterine cavity, a review of which follows a summary of efforts given to retrieving and analyzing fetal cells from maternal blood (FCMB).

### 3.1. Fetal Cells in Maternal Blood

Five cell types have been identified in maternal blood as early as at 8–9 weeks of gestation, but most commonly investigated toward the end of the first trimester and into the second trimester—trophoblasts, granulocytes, lymphocytes, stem cells, and nucleated red blood cells; each of these are at very low concentrations, perhaps one in a billion total cells (which includes erythrocytes in the maternal background). The results of numerous efforts with the goal of establishing a consistent clinical strategy or protocol have been disappointing to date but persistence has been and remains the hallmark of the research. While this research has identified the limitations of FCMB strategies to date, it has also provided insights into the problems yet to be solved. These limitations may be summarized into the following:

- An acknowledged rarity of intact fetal cells in maternal circulation;
- The fragility of target cells that makes delays between blood draw and analysis difficult;
- The relatively low efficiency of enrichment methods leading to loss of fetal samples, sample to sample;
- The possible disintegration of chromosomes before the elimination of the nucleus—specifically from nucleated erythrocytes [19]—thus making FISH potentially unreliable;
- The persistence of white cells from prior pregnancies in maternal circulation for considerable periods of time [20]; and, the difficulty of pinning down markers that are sufficiently distinct or differentially expressed to provide a pointer to a fetal cell time after time.

The majority of strategies and techniques for enriching, isolating, and identifying fetal cells have been reviewed in several publications [3,7,8,9]. These strategies have relied primarily upon a presumed unique fetal identifier in the form of one or more cell surface antigens or cytoplasmic proteins. Antibodies to surface antigens have been linked to substrates to positively select for target cells or used immunohistochemically to highlight a cell against a residual background of maternal cells after enrichment by other means, such as gradient centrifugation and/or depletion of erythrocytes by lysis. Table 1 summarizes the presumed advantages and empirically recognized challenges and disadvantages in the use of leucocytes [21,22,23], trophoblasts [24,25], and fetal red cells [3,7].

The past 7–8 years of continuing efforts have produced both important advances as well as some “red flags”. Using a double Y chromosome FISH strategy to boost identification of fetal cells unselected for cell type, Merganthaler *et al.* [26] concluded that the total number of fetal cells in maternal circulation ranges from 4–36/mL whole blood in contrast to the finding of 2–6 cells/mL reported by Krabchi *et al.* [2] that has served as a common benchmark. However, the Merganthaler group also failed to identify male fetal cells in 43% of the cases later confirmed to be pregnancies with a male fetus, concluding that even a large volume of sampled blood (500 mL maternal blood) may not be sufficient to assure the reliable presence of fetal cells of any type in a non-invasive diagnosis.

**Table 1:** Overview comparisons of trophoblasts, fetal nucleated red blood cells, and fetal leucocytes as sources for prenatal diagnosis.

Cell Type	Comparison	Comments
**Trophoblasts**	Advantages	Large cell size, a distinction demonstrated to be an applicable feature in isolation and analysis
	Disadvantages	Derived from placenta; May be heterogeneous because of multi-nucleation or placental mosaicism; Markers that have not been specific (though recent expression pattern analysis suggests some possibilities)
**nRBC’s**	Advantages	Directly derived from fetus; Short lifespan that should minimize persistence from previous pregnancies
	Disadvantages	Surface markers not sufficiently unique from adult cells; Relatively fragile; Apoptotic processes may lead to inconsistency of analysis; Requires distinction from from pregnancy induced maternal nRBCs
**Fetal Leucocytes**	Advantages	Directly derived from fetus
	Disadvantages	May persist from subsequent pregnancies; Probably rarest of the three cell types in maternal circulation

Despite this finding, several other groups have contributed encouraging as well as important advances. The absence of an antigen or other marker that is sufficiently specific for only fetal cells has probably been the single most limiting factor in any procedure to isolate the rare fetal cell from maternal circulation. Reliance on the presence of the Y chromosome in putative cells can only serve as a marker for proof of principle experiments but must ultimately be replaced by a gender neutral identifier. An encouraging report that used the i-antigen (in combination with other surface antigens) to positively select for both nucleated erythrocytes as well as stem cells (CD34+) [27] may prove to be the most important contribution of a protocol that drew upon other steps that have been used in one combination or another in other previously published research (e.g., Ficoll density centrifugation, bead based depletion of maternal cells, immunocytochemical identification of target cells, FISH analysis of immunostained, presumptive fetal cells). The i-antigen is the fetal precursor to the adult I antigen of the I/i blood group system [28]. The distinction between the two forms is the linearity of the i-antigen galactose-*N*-acetyl glucose repeating unit compared to the branched and longer adult I-form, a conversion mediated by a branching transferase. This conversion, however, has some variability across adults with some individuals having increased levels of the i-antigen with concomitant decreased levels of the I-form, thus posing a potential limitation of its application based on individual differences that may be encountered in pregnant women. Additionally, because immunohistochemical methods involve fixation, recovery of cells for downstream molecular analysis and not limited to FISH poses a continuing challenge that make methodologies that would maintain the cells in solution a strategy with greater versatility of downstream genomic analysis.

The confirmation that a putative fetal cell is indeed fetal has been another layer of problem to be solved in addition to the use of a molecular “finger” to point to the fetal cell initially. For protocols that segregate presumed fetal cells, fingerprinting by STR (short tandem repeat) analysis is a promising technique to distinguish a fetal from maternal cell on a single or set of pooled cells. The use of STR genotyping to assess fetal origin as part of a blind assessment of two single gene mutations was successfully applied by Mouawia *et al.* [25] to the analysis of circulating fetal trophoblasts. This group’s strategy sought to capture cells, one at a time, verify each cell’s identity as fetal (or not) and then analyze individually forming replicates of analysis for reliability of data; this approach mirrors the model strategy described in Figure 1. Because trophoblasts are larger than leukocytes, the ISET method (Isolation by Size of Epithelial Tumor/Trophoblastic cells) captures circulating cells >8 mm on a filter following lysis of erythrocytes and fixation of nucleated cells; the pore size of the filter can be altered to accommodate varying thresholds of exclusion. Capture of retained cells by laser microdissection followed by lysis of each single cell, whole genome amplification, STR genotyping to discriminate fetal from maternal cells, and eventual mutation analysis—in this study, for CFTR (cystic fibrosis transmembrane conductance regulator, associated with cystic fibrosis) mutations and SMN1 (survival motor neuron 1, associated with spinal muscular atrophy, SMA) deletions—resulted in the diagnostic identification of all affected fetuses in the sample (7 of 32 tested for CFTR/cystic fibrosis mutations and 7 of 31 tested for SMN1/spinal muscular atrophy deletions), confirmed later by chorionic villus sampling. Blood samples were obtained between 9 and 11 weeks gestation. In addition, in samples obtained from women who conceived by IVF, circulating fetal cells were captured as early as 4 weeks gestation.

The ability to consistently capture and amplify fetal cells by methods focusing on the use of single cells requires amplification of the genome of one or a few putative fetal cells. While quite powerful, amplification techniques have had limitations of both selectivity across the genome as well as fidelity that is less than perfect thus resulting in amplifications that may be both biased and incomplete. In addition, STR analysis requires that the target fetal cell be disrupted thus driving downstream analysis away from techniques such as FISH and toward genome analyses by techniques such as chromosomal microarray, targeted genotyping, and/or whole genome or exome gene sequencing. Emerging improvements and innovations in amplification strategies may provide the needed solution to providing a consistent genome for both confirmation of identity and, more importantly, a complete diagnostic analysis without reservation as to the integrity of the DNA being evaluated [19,29].

### 3.2. Fetal Cells from the Uterine Cavity

Work from the 1990s [30,31], and recently revisited in [32], looking at the possibility of capturing trophoblasts from areas of the reproductive tract by a minimally invasive procedure has driven a variety of investigations. While studies have not yet defined the timeframe in gestation, or preferred capture technique, that would define the window and protocol of greatest opportunity, it appears that prior to 13–15 weeks, trophoblastic cells reach the uterine cavity after crossing the decidua capsularis [33]. The possibility of analyzing cells captured from this area marks the line of research serving as the most likely alternative to capturing and analyzing fetal cells from the circulation. Isolating fetal cells from the endometrial cavity and/or the cervix as early as five weeks [34] and more practically between 6 and 14 weeks has generated both encouraging results and obstacles to be dealt with. Recent publications and abstracts [35] suggest that recovery and analysis of fetal cells from the uterine cavity still holds some promise.

Samples obtained by transcervical collection methods contain fetal trophoblasts (cyto- and syncytio-), maternal squamous cells, particulate contaminants, blood cells, and spermatozoa. While this highlights that examination of cells’ morphology is important to determine sample contents, more importantly it suggests that the model described in Figure 1 for the retrieval and analysis of fetal cells for diagnostics is as applicable to transcervical retrieval strategies as it is to FCMB.

Just as with fetal cells in circulation, the experience to date points to the need to both differentiate a fetal from non-fetal cell by a specific antigenic marker [35,36,37] using immunohistochemistry or a uniquely expressed RNA using a labeled hybridization probe [38] and then segregate marked cells for analysis individually or in aggregates. Various antibodies to proteins expressed by one or another lineage of cytotrophoblasts have been used or suggested—e.g., HLA-G, PLAC-1, GATA2/3, NDOG1. The value of having one or more identifiers is underscored by the data from Sifakis *et al.* [39] who based their protocol on the premise that a finding of trisomy in cervical cells was equivalent to a fetal cell identifier. While this may be true for studies seeking only to identify fetal aneuploidy, it is not generalizable to the diagnosis of subchromosomal and gene level alterations. Additionally, one cell from the 28 first trimester control samples in this study was XX + 21, calling attention to both the limitation of an aneuploidy finding as a proxy for a fetal cell as well as the ultimate need to have a statistical basis for a minimal number of analyzed cells from any single sample to make a diagnostic call.

Techniques for collecting cells have included cotton swabs, cytobrush, aspiration by catheter of cervical mucus, biopsy of the endometrium, and lavage of the endocervical canal or uterine cavity. Intrauterine lavage methods appear to have been more successful than mucus collection as measured by accurate and predictive analysis of X and Y chromosomes of retrieved cells. A comparison of four methods [32] for the collection of cells from the reproductive tract points to a higher yield using a cytobrush than three other methods surveyed—endocervical canal lavage, intrauterine lavage, aspiration of cervical mucus, and flushing of the endocervical canal with saline. As these authors pointed out, there is a considerable degree of operator dependence in the absence of well described sampling techniques to establish a grounded basis for comparisons [32]. However, reports that retrieval can be as high as 95%–97% of samples using a cytobrush technique [40,41] is an encouraging direction for future efforts. Recent reports by Sinosich [35] point to successful retrieval of terminally differentiated syncytiotrophoblasts containing up to 50 nuclei following specific identification using a combination of monoclonal antibodies (not specified). The technique for retrieval is not clear from the abstract describing this work but further studies may demonstrate the clinical validity of the claims as well as the scope of downstream analyses.

There are several unresolved questions to date as studies continue to pursue the retrieval of fetal cells from the uterine canal. As noted above, the post-collection processing and analyses has pointed to a need for cell differentiating strategies as well as techniques to address confounding characteristics specific to this type of sample (e.g., use of mucolytics, fixation *vs.* maintaining cells in solution). While there are no formal studies comparing the two approaches, there is little dispute that lavage is considered to be more invasive than cytobrush and therefore implies a higher risk of infection. If the presence of polyploidy in a small fraction of recovered trophoblasts [42] will have any meaning to downstream analysis along with questions of whether the use of trophoblastic cells generally will encounter the confounding data produced by placental mosaicism as is known for cells obtained by CVS is a question remaining to be worked through. In principle, cells collected transcervically should provide extensive information about the fetal genome at the chromosomal level to the single or multiple gene levels.

## 4. Where to from Here?

Perhaps the most important set of questions that will need answering in the coming years—beyond the basic ones that would lead to a cost-effective and reliable isolation followed by sensitive and accurate analyses—are those that are generated when comparing the current and future capacities of cell-free DNA analysis with fetal cell-based techniques; some of these are captured in Table 2 as comparisons of the distinctive as well as common features that fetal cells and fetal (placental) cell-free DNA.

**Table 2:** Some distinctive and common qualities in the comparison of fetal cell and cell free DNA based prenatal testing.

Question	DNA Source	Distinctive Qualities	Common Qualities
**What are the instrument needs?**	Fetal Cells	Cell sorting Microscopes Cell culture (?) CGH-SNP Platform	PCR related NextGen Sequencing
	Fetal Cell-Free DNA	DNA isolation Mass spectrometer	
**What are the advantageous capabilities?**	Fetal Cells	Potential to be a diagnostic test and not limited to screening Direct analysis of single (?) or pooled (?) cells using biological measurements Amenable to FISH and/or qfPCR analyses for rapid analysis of aneuploidy Capacity for single gene analysis, variation screening or sequencing Potential for functional and polygenic analyses Amenable to CNV (copy number variation) determinations by CGH-SNP analysis	Non-invasive Capacity for both aneuploidy and CNV analyses
	Fetal Cell-Free DNA	Preparation more rapid than cells Minimal problems in transporting blood from clinic to centralized labs Potential for CNV determinations by deep sequencing and analysis	
**What are the apparent disadvantages or important challenges to be met?**	Fetal Cells	Isolation of cells may be labor intensive; cost effective throughput has not been demonstrated Integrity of DNA in possibly apoptotic cells may dictate consistency and quantity of cells required for reliable evaluations Requires faithful and complete amplification of DNA Likely to be few cells analyzed from a sample and thus less likely to be representative of a mosaic condition Stability and integrity maintenance requirements for transport of cells from phlebotomist to laboratory	Requisite equipment and expertise may limit distribution beyond centralized laboratories
	Fetal Cell-Free DNA	Becoming validated as an effective diagnostic as well as screening test Accuracy seems to depend on level of proportion of extracted DNA derived from fetus Results are based on (powerful) statistical methods rather than direct biological measurements Unable to determine mosaicism if present Degree of extension of analyses beyond aneuploidy yet to be determined and validated	

The clinical future of fetal-cell based diagnosis lies with the development of a reliable, relatively rapid, and cost effective system that is at least applicable to all women who would seek prenatal information. Currently, analysis of direct CVS samples can provide a same to next day assessment of fetal aneuploidy by FISH with confirmation to follow within 4–5 days that is extensible to CNV analysis by microarrays [43]. Diagnostic analysis of cultured CVS or amniotic fluid cells can occur within in 7–10 days post-procedure, depending on the extent of cell culturing needed. Additionally, CVS and/or amniocentesis samples can be transported with relative ease from the clinician’s office to a laboratory even at some distance. The adoption of prenatal diagnosis by fetal cells obtained non-invasively would exist within this context. The third perspective, described earlier in this review, leads to the question of whether the future of fetal cell based prenatal diagnostics is only a technological advance away or whether the difficulties that have been persistently encountered mark a biology of fetal-maternal trafficking that requires further knowledge and insights before solutions can be devised. The answers may lie with both.

Once the absolutely critical requirement of identifying a fetal cell consistently and reliably is met, some of the answers may lie in the transfer of strategies from one arena of cell biology and molecular biology, or even non-biological realms—for example, from investigations of circulating tumor cells as has already been or applied [38,44], from highly advanced cell sorting techniques (e.g., DEParray [45]); from improvements in genome amplification (e.g., multiple annealing and looping based amplification, MALBAC [46]); from the application of cell-free DNA sequencing techniques to maternal blood fractions enriched with fetal cells; from computational strategies for assessing rare events [47] and detecting rare phenomena, which has been an analogous problem for astronomers seeking to capture rare astronomical events [48].

Is it technology or biology? Whether progress has been hampered because the biology of fetal cells and maternal-fetal trafficking imposes natural limitations on the consistent identification and isolation of fetal cells for consistent clinical application or the right technology has not been yet developed and/or been brought together to accomplish the goal of non-invasive prenatal diagnosis using fetal cells is not yet clear. The eventual answer(s) may simply come from clinical trials of methods that address the essential elements outlined in Figure 1. Such trials would presumably capture data on nagging questions, such as: Will microchimeric engraftments associated with prior pregnancies interfere with an analysis of a current pregnancy [20] and/or might there be strategies to anticipate when this will be of significance for each individual? What is the true level of variation that exists from pregnancy to pregnancy in the level of fetal cells in circulation and what are the implications of such variation for the universality of a fetal based diagnostic strategy? Is the DNA from cells that have made their way from the fetus to maternal circulation of sufficient integrity to be interrogated by existing techniques? 

The development of cfDNA testing has established a new model for prenatal testing that brings it closer to a routine clinical protocol of drawing blood or collecting a urine sample and sending it off to a centralized laboratory for processing and workup. Presumably, the model for delivering fetal cell based prenatal testing will be dictated by whatever the successful technology(ies) turn out to be. Advances and applications in prenatal diagnosis have, until recently, been in a decentralized environment of skilled practitioners developing, improving and performing procedures and working with skilled laboratorians providing analyses and improving upon these as needed as well; sometimes, these have been one and the same. It is difficult to foresee a cell based methodology that does not require highly specialized equipment and skills but also is not relatively labor intensive. Questions of scalability and quality assurance are inevitable; the former would be particularly highlighted by a centralized model for carrying out analyses and the latter accented should the technology be employed on a decentralized, disseminated basis. The decisions on these matters are likely to have impacts on the collective contribution of the medical scientific community to ongoing validation, problem identification and solving, incremental improvements, and progressive establishment of standards based on diverse experiences.

## 5. Conclusions

There is still a need for more technological development and clinical trials before the use of fetal cells can be fully assessed and applied to clinical medicine. Will cell based prenatal analyses eventually meet or exceed the recent successes of cell free DNA testing as a screening strategy or become validated as a diagnostic technique? Or might the use of fetal cells become an intermediary strategy for women identified first as being at-risk by cfDNA testing to be then followed by fetal cell based testing with optional final confirmation by an invasive procedure? This scenario seems unlikely. It is more likely that fetal cell based prenatal diagnosis will become an “all or nothing” presence in the repertoire of prenatal diagnostics. The unasked or unrealized questions as well as the answers to the current questions rest in the significant work that still lies ahead.
